# Near-Complete Genome Sequences of a *Wolbachia* Strain Isolated from Diaphorina citri Kuwayama (*Hemiptera*: *Liviidae*)

**DOI:** 10.1128/MRA.00560-20

**Published:** 2020-08-27

**Authors:** Surendra Neupane, Sylvia I. Bonilla, Andrew M. Manalo, Kirsten S. Pelz-Stelinski

**Affiliations:** a Entomology and Nematology Department, Citrus Research and Education Center, University of Florida, Lake Alfred, Florida, USA; Indiana University, Bloomington

## Abstract

*Wolbachia* strains are one of three endosymbionts associated with the insect vector of “*Candidatus* Liberibacter asiaticus,” Diaphorina citri Kuwayama (*Hemiptera*: *Liviidae*). We report three near-complete genome sequences of samples of *Wolbachia* from *D. citri* (wDi), with sizes of 1,518,595, 1,542,468, and 1,538,523 bp.

## ANNOUNCEMENT

The widespread endosymbiont *Wolbachia* is present in many insect species and can manipulate host reproduction via cytoplasmic incompatibility, male killing, and induction of parthenogenesis or feminization (1). Diaphorina citri Kuwayama (*Hemiptera*: *Liviidae*), the vector of the pathogen “*Candidatus* Liberibacter asiaticus,” associated with citrus greening disease, harbors three intracellular symbionts, including a strain of *Wolbachia*, wDi. Currently, the functional relationship of *Wolbachia* sp. strain wDi with *D. citri* and “*Ca.* Liberibacter asiaticus” is limited due to the unavailability of a genome assembly with no gaps. Here, we report three wDi genome sequences utilizing both long- and short-read sequencing methods. *Wolbachia* isolates were recovered from *D. citri* from an established laboratory culture collected in Polk County (28.0′N, 81.9′W; Lake Alfred, FL, USA). Individual psyllids were placed on sterile diet rings for 2 days prior to *Wolbachia* extraction. The psyllids were surface sterilized and immersed in 1.0 ml of Schneider’s *Drosophila* (S2) medium (catalog number 21720024; Gibco). Next, individual psyllids were homogenized and centrifuged at 100 × *g* for 5 min. The supernatants were collected and centrifuged at 400 × *g* for 5 min. The pellets were resuspended with 1.0 ml of S2 medium. The samples were centrifuged at 100 × *g* for 5 min; then, supernatants were placed in new tubes and centrifuged at 4,000 × *g* for 5 min. The pellets were resuspended in 1.0 ml of S2 medium. After isolation, an individual wDi sample was inoculated into *Drosophila* S2 cells (catalog number R69007; Gibco) and maintained in S2 medium containing 10% heat-inactivated fetal bovine serum (catalog number 16000044; Gibco), 50 units of penicillin, and 50 μg streptomycin sulfate (catalog number 15070-063; Gibco) per ml (S2 complete medium) using the method described by Dobson et al. (2). wDi-infected S2 cells were maintained using standard protocols (3). *Wolbachia* isolates were purified from a parental cell line at different passages (28, 36, and 40) to detect the generic variation of the bacteria in the host, using the protocol adopted from Rasgon et al. (4). The genomic DNA from three wDi samples were extracted using the MagAttract high-molecular-weight (HMW) DNA minikit (catalog number 67563) using the manufacturer’s protocol with little modification. The extracted DNA samples from passages 28 and 40 were cleaned up using the DNeasy PowerClean cleanup kit (catalog number 12877-50). The DNA sample from passage 36 was left unpurified. The extracted DNA was quantified using the Qubit 1× double-stranded DNA (dsDNA) high-sensitivity (HS) assay kit (Thermo Fisher Scientific), and the quality was measured using the TapeStation genomic DNA ScreenTape (Agilent). For PacBio sequencing, a library was prepared using single-molecule real-time (SMRT) sequencing in conjunction with barcodes from the barcode adaptor kit 8A (PacBio PN 101-081-300). Sequencing of wDi was done using a PacBio SEQUEL instrument using chemistry v3.0 reagents in combination with the SMRTLink v6.0 software. For Illumina sequencing, the libraries were prepared using the NEBNext Ultra II DNA library prep kit (New England BioLabs, Ipswich, MA, USA), and genomic sequencing was performed using a MiSeq instrument (Illumina, San Diego, CA) with the 2 × 300-bp paired-end protocol using the 600-cycle v3 kit (Illumina).

**FIG 1:**
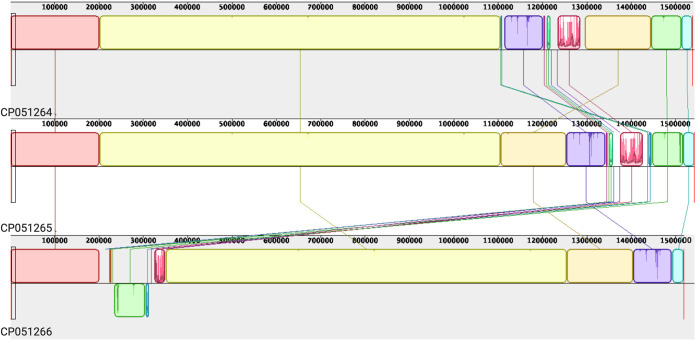
Mauve alignments showing chromosomal similarity among the genome assemblies under GenBank accession numbers CP051264, CP051265, and CP051266. Ten local colinear blocks (LCBs) on the chromosomes were identified and joined by connecting lines in the three genomes. Two LCBs in the assembly under accession number CP051266 are inverted, which shows reverse complement orientation.

The circular consensus sequences (CCS) generated from the PacBio raw reads were subjected to quality assessment and adaptor trimming using seqtk (https://github.com/lh3/seqtk) and Filtlong (https://github.com/rrwick/Filtlong). *De novo* assembly was performed separately using Canu v1.9 (5), Unicycler v0.4.8 (6), and the hierarchical genome assembly process (HGAP) (7). These assemblers produced different-sized contigs, which were subassembled using the subassemblies parameter in Flye v2.7.1 (8) to produce the longest contig (wDi from passages 28 and 36). Canu produced the best assembly for wDi from passage 40; however, the genome sequences could not be circularized using the available tools. Paired-end Illumina reads were cleaned with Trimmomatic v0.36 (9) to remove adapters, and sequences with a Phred score of less than 30 were discarded. The trimmed Illumina reads were mapped to the corresponding PacBio chromosome using bwa v0.7.17 (10) using paired-end mode. Indel correction and polishing were done using Pilon v1.22 (11). The Pilon analyses corrected 13 single nucleotide polymorphisms (SNPs), 66 small insertions totaling 74 bases, and 1 small deletion totaling 1 base in wDi from passage 28 and 206 SNPs and 103 small insertions totaling 135 bases and 32 small deletions totaling 97 bases in wDi from passage 36. The Pilon tool did not correct any bases in wDi from passage 40. Genome annotation was conducted using the standard NCBI Prokaryotic Genome Annotation Pipeline (PGAP) (12). Assembly statistics and general genome information for the three wDi genome sequences are represented in Table 1. Mauve (13) was used for wDi genome alignment with local colinear blocks (LCBs). Ten LCBs on each chromosome were identified and joined by connecting lines in the three genome sequences (Fig. 1).

**TABLE 1:** Assembly statistics and general genome information of three wDi genome sequences

Parameter	Data for isolate:		
	wDi (passage 28)	wDi (passage 36)	wDi (passage 40)
Sequencing instrument	PacBio Sequel II	PacBio Sequel II	PacBio Sequel II
No. of polymerase reads	79,755	113,497	130,351
No. of subreads	868,919	1,298,934	1,534,487
No. of bases	4,421,085,394	6,263,217,203	7,244,262,525
Mean read length (bp)	56,224	56,019	56,435
Longest subread length (bp)	182,617	127,315	162,373
No. of CCS bases	186,807,190	257,167,133	294,137,324
No. of CCS reads	35,203	51,403	59,780
CCS coverage (×)	124.53	171.44	196.09
Assembled chromosome length (bp)	1,518,595	1,542,468	1,538,523
G+C content (%)	34.06	33.95	33.92
No. of genes (total)	1,415	1,428	1,409
No. of CDSs^*a*^ (total)	1,376	1,387	1,368
No. of CDSs^*a*^ (with protein)	1,251	1,249	1,238
No. of RNAs	39	41	41
No. of rRNAs	3	3	3
No. of tRNAs	32	34	34
No. of ncRNAs^*b*^	4	4	4
No. of pseudogenes (total)	125	138	130
CheckM completeness (%)	95.7	100	100
CheckM contamination (%)	0.43	0.43	0.43
PacBio accession no.	SRR11513698	SRR11513697	SRR11513696
Illumina accession no.	SRR11513759	SRR11513758	SRR11513757
GenBank accession no.	CP051266	CP051265	CP051264

a CDSs, coding DNA sequences.

b ncRNAs, noncoding RNAs.

Benchmarking Universal Single-Copy Orthologs (BUSCO) v4 using the bacteria_obd10 database (14) was utilized to assess the completeness of the genomes. The completeness rates of the final assembled genomes were 80.6% (wDi sample from passage 28) and 85.5% (wDi samples from passages 36 and 40), compared with those of the reference *Wolbachia* genome sequences, wAlbB (from Aedes albopictus) (84.7%) (15), wMel (from Drosophila melanogaster) (83.1%) (16), and *Wolbachia* sp. strain wPip (from Culex pipiens) (86.3%) (17). In addition, CheckM analysis (18) was done to assess the completeness and contamination using 63 genomes and 368 lineage-specific markers via the Microscope platform (19) (Table 1). The genome assemblies were examined for prophage sequences using PHAge Search Tool Enhanced Release (PHASTER; https://phaster.ca/) (20). PHASTER predicted variations in the three assemblies, with seven regions (range, 11.7 kb to 31.5 kb) in wDi from passage 28, six regions (range, 11.8 kb to 35.7 kb) in wDi from passage 36, and five regions (range, 11.8 kb to 35.3 kb) in wDi from passage 40.

### Data availability.

The three genome sequences of wDi have been deposited in GenBank under the accession numbers CP051266, CP051265, and CP051264 corresponding to BioSample accession numbers SAMN14560310, SAMN14560311, and SAMN14560312, respectively, under the BioProject accession number PRJNA603775.
